# Cardiac Derived CD51-Positive Mesenchymal Stem Cells Enhance the Cardiac Repair Through SCF-Mediated Angiogenesis in Mice With Myocardial Infarction

**DOI:** 10.3389/fcell.2021.642533

**Published:** 2021-04-21

**Authors:** Dong Mei Xie, Yang Chen, Yan Liao, Wanwen Lin, Gang Dai, Di Han Lu, Shuanghua Zhu, Ke Yang, Bingyuan Wu, Zhihong Chen, Chaoquan Peng, Mei Hua Jiang

**Affiliations:** ^1^Department of Cardiology, The Third Affiliated Hospital, Sun Yat-sen University, Guangzhou, China; ^2^Department of Anatomy, Zhongshan School of Medicine, Sun Yat-sen University, Guangzhou, China; ^3^Key Laboratory for Stem Cells and Tissue Engineering, Center for Stem Cell Biology and Tissue Engineering, Ministry of Education, Sun Yat-sen University, Guangzhou, China; ^4^Shenzhen Beike Biotechnology Co., Ltd., Shenzhen, China; ^5^NHC Key Laboratory of Assisted Circulation, Sun Yat-sen University, Guangzhou, China; ^6^Department of Anesthesiology, The First Affiliated Hospital, Sun Yat-sen University, Guangzhou, China

**Keywords:** CD51, mesenchymal stromal/stem cells, myocardial infarction, angiogenesis, stem cell factor (SCF)

## Abstract

**Objective:** Many tissues contained resident mesenchymal stromal/stem cells (MSCs) that facilitated tissue hemostasis and repair. However, there is no typical marker to identify the resident cardiac MSCs. We aimed to determine if CD51 could be an optimal marker of cardiac MSCs and assess their therapeutic potential for mice with acute myocardial infarction (AMI).

**Methods:** Cardiac-derived CD51^+^CD31^–^CD45^–^Ter119^–^ cells (named CD51^+^cMSCs) were isolated from C57BL/6 mice(7-day-old) by flow cytometry. The CD51^+^cMSCs were characterized by proliferation capacity, multi-differentiation potential, and expression of typical MSC-related markers. Adult C57BL/6 mice (12-week-old) were utilized for an AMI model via permanently ligating the left anterior descending coronary artery. The therapeutic efficacy of CD51^+^cMSCs was estimated by echocardiography and pathological staining. To determine the underlying mechanism, lentiviruses were utilized to knock down gene (stem cell factor [SCF]) expression of CD51^+^cMSCs.

**Results:** In this study, CD51 was expressed in the entire layers of the cardiac wall in mice, including endocardium, epicardium, and myocardium, and its expression was decreased with age. Importantly, the CD51^+^cMSCs possessed potent self-renewal potential and multi-lineage differentiation capacity *in vitro* and also expressed typical MSC-related surface proteins. Furthermore, CD51^+^cMSC transplantation significantly improved cardiac function and attenuated cardiac fibrosis through pro-angiogenesis activity after myocardial infarction in mice. Moreover, SCF secreted by CD51^+^cMSCs played an important role in angiogenesis both *in vivo* and *in vitro*.

**Conclusions:** Collectively, CD51 is a novel marker of cardiac resident MSCs, and CD51^+^cMSC therapy enhances cardiac repair at least partly through SCF-mediated angiogenesis.

## Background

Myocardial infarction (MI) accounts for 80% of death in patients with ischemic heart disease worldwide (Terzic and Behfar, 2016). Owing to the contemporary management methods, the rates of short-term survival increased in patients with acute MI (AMI) (Anderson and Morrow, 2017). Particularly, the substantial decline in case fatality rates among AMI patients is closely related to the application of revascularization strategies, such as percutaneous techniques, coronary artery bypass grafting, intravenous fibrinolytic therapy, antithrombotic and anticoagulant agents (Neumann et al., 2019). However, many surviving patients subsequently develop heart failure, which is attributed to long-term hospitalization and death (See et al., 2017). As a key part of the cardiovascular system, the heart needs to be enriched with blood supplement to keep beating, and it receives its own blood supplement from a network of arteries and their branches. Impaired microcirculation predicts poor outcome of patients with AMI (den Uil et al., 2010). Therefore, innovative opportunities to enhance cardiac microcirculation are demanded to protect the heart tissues from injury. Stem cell therapy is being investigated as a promising strategy to regenerate the damaged myocardium and restore cardiac function in patients with ischemic heart disease.

Mesenchymal stromal/stem cells (MSCs) are multipotent progenitors, which were isolated from the bone marrow or other tissues. Previous studies have shown that MSCs hold great potential for cardiovascular disease because of their feasibility, safety, and efficiency (Horwitz et al., 2005; Keating, 2006; Hare et al., 2012; Karantalis et al., 2015). Although the molecular mechanism for the function of MSCs in facilitating engraftment and accelerating heart functional recovery is still unclear, the therapeutic efficacy of MSCs has increasingly been attributed in clinical trials (Wang et al., 2012; Wollert et al., 2017). Importantly, the characteristics of MSCs are significantly different depending on their origin and therapeutic capability for cardiac vascular disease (Gomes et al., 2013; Karantalis et al., 2015; Gavhi et al., 2020). However, numerous studies indicate that the success of the field will likely require our understanding of developmental biology and the role of the different types of MSCs, in order to refine their manufacturing and maximize their capacity to promote repair (Amable et al., 2014; Heldman et al., 2014; Hare et al., 2017). Accordingly, cardiac resident stromal cell-based therapy is a promising strategy for the treatment of ischemic heart disease, but isolating these cells is still difficult because of the lack of specific markers, which impeded the recognition and application of resident cardiac MSCs (Bianco et al., 2008). Integrin alpha ν (CD51), a heterodimeric integral membrane protein, is composed of three domains: an extracellular domain, a transmembrane region, and a cytoplasmic domain (Hynes, 1992; Sosnoski et al., 1988). Local expression of CD51 increased during the early stage after injury, and it helped to repair damaged tissues (Breuss et al., 1995). Sandra Pinho et al. reported that a human bone marrow CD51^+^PDGFRα^+^ MSC subpopulation could transform into multipotent hematopoietic stem or progenitor cells and expressed abundant hematopoietic stem cell maintenance genes (Pinho et al., 2013). Furthermore, CD51 was detectable in other MSCs isolated from the bone marrow, testis, umbilical cord blood, periodontal tissue, and craniofacial tissues (Thoma et al., 1994; Wang et al., 2004; Pinho et al., 2013; Jiang et al., 2014; Alvarez et al., 2015; Zang et al., 2017; Xie et al., 2019). Thus, we hypothesized that CD51 may be a crucial marker of cardiac resident MSCs.

In this study, we initially analyzed CD51 expression in hearts and evaluated the feasibility that CD51 served as a surface marker to identify MSCs from hearts of postnatal mice. Next, we assessed the therapeutic efficiency of CD51^+^cMSC transplantation through testing heart function and histological staining cardiac tissues. Finally, we explored the underlying mechanism and confirmed the candidate paracrine function both *in vivo* and *in vitro*.

## Materials and Methods

### Animals

C57BL/6 mice were purchased from the Animal Center of Medical Laboratory of Guangdong Province and were fed in microisolator cages under specific pathogen-free conditions with an ambient temperature of 24°C, 55–65% relative humidity, and a 12:12 h light:dark cycle. Adult male C57BL/6 mice (12-week-old) were used for *in vivo* experiments, and they were randomly allocated to each group (12 mice per group). All animal protocols were reviewed and approved by the Sun Yat-sen University Institutional Animal Care and Use Committee.

### Isolation and Culture of Cells From the Hearts of Neonatal Mice

The hearts of 7-day-old C57BL/6 mice were harvested and cut into pieces and then incubated with 5 ml HBSS digestion solution containing type II collagenase (300 U/ml; Gibco) and DNase I (100 U/ml; Sigma Aldrich, United States). After the tissues were homogenized, the homogenate was incubated at 37° for 30 min with shaking every 10 min. Subsequently, the cell suspensions were passed through a 40 μm cell strainer, collecting single cells. Thereafter, single cells were resuspended and incubated with flow cytometry antibodies at 4°C for 30 min. CD51^*pos*^, CD51^*neg*^, and CD51^+^CD45^–^Ter119^–^CD31^–^ cells were sorted using flow cytometry (Influx, BD, United States). The antibodies used in this study were described as follows: PI (dilution 1:200, Invitrogen, United States), CD51-PE (dilution 1:200, BD, United States), CD45 (dilution 1:200, eBioscience, United States), Ter119 (dilution 1:200, eBioscience), CD31 (dilution 1:200, eBioscience), and CD140a-APC (dilution 1:200; BD Biosciences). The isolated cells were cultured with a 2:1 mixture of DMEM/F12 (Gibco, United States) and IMDM (Gibco) (2:1) supplemented with 25 ng/ml EGF (PeproTech, United States), 20 ng/ml bFGF (PeproTech), 1% N-2 (Invitrogen, United States), 2% B-27 (Invitrogen), 4 ng/ml cardiotrophin 1 (PeproTech), 0.1 mM β-mercaptoethanol (Invitrogen), 1% L-glutamine (Sigma Aldrich, United States), 1% fetal bovine serum (Gibco), and 100 IU/ml penicillin/streptomycin (Invitrogen). Cells were cultured at 37°C in a 5% CO_2_ atmosphere, and propagated every 3 days.

### Clone Formation Assays Using Freshly Sorted CD51^*pos*^ Cells and CD51^*neg*^ Cells

As recommended by Phuc Van Pham, one cell sorted into one well of a 96-well plate is a good strategy to test clone formation capacity of stem cells (Phuc Van Pham, Books, Biomedical Tissue Culture). This strategy ensured a colony was replicated through one viable cell, but not a mass of deposited cells. One fresh CD51^*pos*^ cell or CD51^*neg*^ cell was sorted into one well of an untreated 96-well plate containing the culture medium described above and then incubated at 37°C with 5% CO_2_, following half-volume medium refresh every day. After 7 days of culture, the replicated colony-like structures were calculated through an optical microscope. Colony-like structures with three or more cells were considered positive, and the positive ratio was calculated as follows: (number of colony-like structures/96 inoculated) × 100%.

### Flow Cytometry Analysis

Expression of surface markers or green fluorescent protein (GFP) in the cultured CD51^+^cMSCs was tested using Influx flow cytometers (BD, United States). Assessment of a CD51-positive subpopulation with other subpopulations (Sca-1/C-kit/CD31/CD45/Ter19) in the heart tissue was also analyzed using flow cytometry. Data were analyzed with FlowJo 7.5 software (Treestar, United States). The following anti-mouse antibodies were used: CD51-PE (BD), Ter119-PE Cyanine 7 (eBioscience), CD44-PE (eBioscience), CD106-Alexa Fluor 647 (eBioscience), CD90 (BD Biosciences), Sca-1-APC (eBioscience), C-kit-APC-eFluor 780 (eBioscience), CD34-FITC (eBioscience), and CD11b-APC (eBioscience). The antibodies were used at the dilutions recommended by the manufacturers.

### Proliferation Assays

CD51^+^cMSCs (passages 5, 15, and 25) were resuspended in the medium and seeded in 24-well plates at a density of 3 × 10^4^ cells per well. Cells were harvested at the indicated time points for 5 days, and cell numbers were directly counted under the microscope (three replicates per sample).

### Cell Differentiation *in vitro*

To investigate the multi-lineage differentiation ability, CD51^+^cMSCs were cultured in the conditioned medium to induce osteogenesis, adipogenesis, and chondrogenesis (Cyagen, MUBMX-90021, MUCMD-90031, and MUBMX-9004, respectively) for 2–3 weeks. Then, differentiation cells were stained with Alizarin Red, Oil Red O, and toluidine blue. In addition, specific gene expressions of differentiated osteocytes, adipocytes, and chondrocyte were tested by qPCR.

### Establishment of the AMI Model and Cell Transplantation

As previously reported, the AMI model in mice (12-week-old) was performed by permanently ligating the left anterior descending coronary artery, 0.3 cm from the origination (Muthuramu et al., 2014). After the ischemic area turned pale, 25 μl suspension containing 3 × 10^5^ CD51^+^cMSCs was injected into the infarcted border zone of the myocardium, and injection of same-volume saline was considered a vehicle-treated control. Then, the incision was sutured, and mice recovered and were fed as described above.

### Echocardiography Analyses of Heart Function

To determine the therapeutic effect of CD51^+^cMSCs in mice, we evaluated heart functions, the infarct size, and the survival rate in a blinded fashion. At 1 and 4 weeks after AM, conventional echocardiography of the left ventricle (LV) was performed with a mouse echocardiography system (Vevo 2100 Imaging System, Visual Sonics, Toronto, Canada) equipped with a 30 MHz phased transducer. The following parameters were measured: LV ejection fraction (LVEF), LV fractional shortening (LVFS), LV end systolic volume (LVESV), and LV end diastolic volume (LVEDV). The survival time of the experimental mice was recorded for 30 days.

### Histological Analysis

For immunofluorescence staining, hearts were fixed with 4% paraformaldehyde, dehydrated in 30% sucrose, and then cut into 5 μm sections. The fixed cultured cells and heart tissue sections were blocked with 30% normal goat serum for 40 min, incubated with primary antibodies overnight at 4°C, and subsequently incubated with secondary antibodies (1:500 dilution) in the dark at room temperature for 1 h. The following primary antibodies were used: anti-CD51 (1:100, Abcam, United Kingdom), Sca-1 (1:50, Santa Cruz, United States), C-kit (R&D, United States), anti-CD31 (1:200, Abcam), and anti-Ki67 (1:200, Abcam). For immunohistochemistry, hearts were fixed overnight with 10% formalin and then embedded in paraffin. The processed paraffin slides (5 μm) were blocked with normal goat serum, incubated with a monoclonal anti-CD31 antibody (1:200, Abcam) overnight at 4°C, developed in DAB, and counterstained with hematoxylin according to the manufacturer’s instructions. Endothelialization was calculated as the ratio of the surface covered by the number of vessels stained by CD31. To evaluate the infarct size, paraffin sections of hearts (4 weeks post MI) were subjected to Masson’s trichrome staining (Servicebio, China).

### Reverse-Transcription and Real-Time Quantitative PCR

The total RNA was extracted from cultured cells or heart tissues with TRIzol reagent (Invitrogen, Carlsbad, CA). RNA (1 μg) was reverse transcribed using the RevertAid First-Strand cDNA Synthesis Kit (Thermo Scientific). The cDNA templates were subjected to a real-time quantitative PCR (qPCR) procedure with the SYBR Green reagent (Roche, Indianapolis, United States) and the listed primers (Supplementary Table 1). The relative mRNA abundance was calculated using the ΔCt method, and gene expression levels were normalized to GAPDH.

### Construction and Transfection of the Lentiviral Vector for RNA Silencing

The short hairpin RNA (shRNA) targeting the mouse stem cell factor (SCF) gene was designed and synthesized by Sangon Biotech (Shanghai, China). The following sequences were used: forward TGATATGATAACCCTCAACTATGCTTCCTGTCACATAGTT GAGGGTTATCATATTTTTTTC and reverse TCGAGAA AAAAATATGATAACCCTCAACTATGTGACAGGAAGCATA GTTGAGGGTTATCATATCA. The lentiviral vector LentiLox 3.7 (pLL3.7) was used to infect and efficiently silence SCF expression in CD51^+^cMSCs (named CD51^+^cMSC^*shSCF*^), and an insert-free vector was used as a negative control (named CD51^+^cMSC^*con*^). Cells were cultured in six-well plates at a density of 5 × 10^4^ cells per well overnight. After refreshing the medium, the mouse lentivirus (multiplicity of infection [MOI] = 10) carrying the GFP reporter gene was added in cells and incubated for 24 h. GFP expression was analyzed by flow cytometry at 72 h after infection. The interference efficiency of SCF was confirmed using a qPCR array and western blot experiment.

### Western Blot Analysis to Determine the Efficiency of SCF Interference

Total proteins were extracted from cells using RIPA lysis buffer, and the concentrations were measured by a BCA protein assay kit (Thermo Scientific, Rockford, AL). Proteins were electrophoretically separated on 8% and 10% sulfate-polyacrylamide gels and then transferred to polyvinylidene fluoride membranes. Next, membranes were blocked in 0.1% Tween Tris-buffered saline (TBS) containing 5% non-fat dry milk for 1 h at room temperature. Then, membranes were incubated with primary antibodies (SCF, R&D Systems; β-actin, CST) overnight at 4°C, following with corresponding secondary antibodies incubation for 1 h at room temperature. Finally, the target protein signals were visualized by chemiluminescence substrate.

### Tube Formation and Cell Proliferation Assays

As for the tube formation experiment, cardiac-derived endothelial cells (1 × 10^4^/ml) were seeded in Matrigel-coated 24-well plates for 2 h, and then the EGM medium was replaced by the CD51^+^cMSC culture medium. Mock-transfected CD51^+^cMSC^*con*^ and SCF-silenced CD51^+^cMSC^*shSCF*^ (3 × 10^4^/ml) were seeded in the upper well of the Transwell inserts and cocultured for 48 h under the hypoxia condition (1% O_2_). Tube formation was measured under a microscope. For the cell proliferation assay, the coculture system was similar, but the Matrigel was not used. Endothelial cells were stained with Ki67 antibody and 4′,6′-diamidino-2-phenylindole (DAPI). Five images per experiment were included in the statistical analysis.

### Migration Assay

Effects of designated cells on migration ability of endothelial cells were determined using a 24-well Millicell hanging cell culture insert (8 μm pore size membrane filters, Merck, United States). First, the lower chamber of the 24-well plate was filled with 600 μl cell suspension containing CD51^+^cMSC^*con*^, CD51^+^cMSC^*shSCF*^, and young CD51^+^ cells. Then, 100 μl endothelial cell suspension (5 × 10^5^ cells/ml) in serum-free medium was plated into the rehydrated upper chamber. The cells were incubated for 8 h under hypoxia condition (1% O_2_). Thereafter, the inserts were fixed in 4% paraformaldehyde for 20 min and stained with crystal violet for 30 min or with DAPI for 5 min. Images of the cells on the lower surface of the filters were acquired by fluorescence and bright-field microscopy (BioTek, United States). The migrated cells were quantified in five randomly selected fields.

### Statistical Analysis

All results were obtained from at least three independent experiments and presented as the means ± SEM. Most statistical comparisons were performed using two-tailed Student’s *t*-test (between two groups) or a one-way ANOVA (for multi-group comparisons). Survival curves were compared using the log-rank test. ^∗^*P* < 0.05, ^∗∗^*P* < 0.01, and ^∗∗∗^*P* < 0.001 were considered statistically significant. Analyses were performed, and graphs were generated using the GraphPad Prism software 6.01 (San Diego, CA).

## Results

### Characteristics of Cardiac CD51 in Mice

**FIGURE 1:**
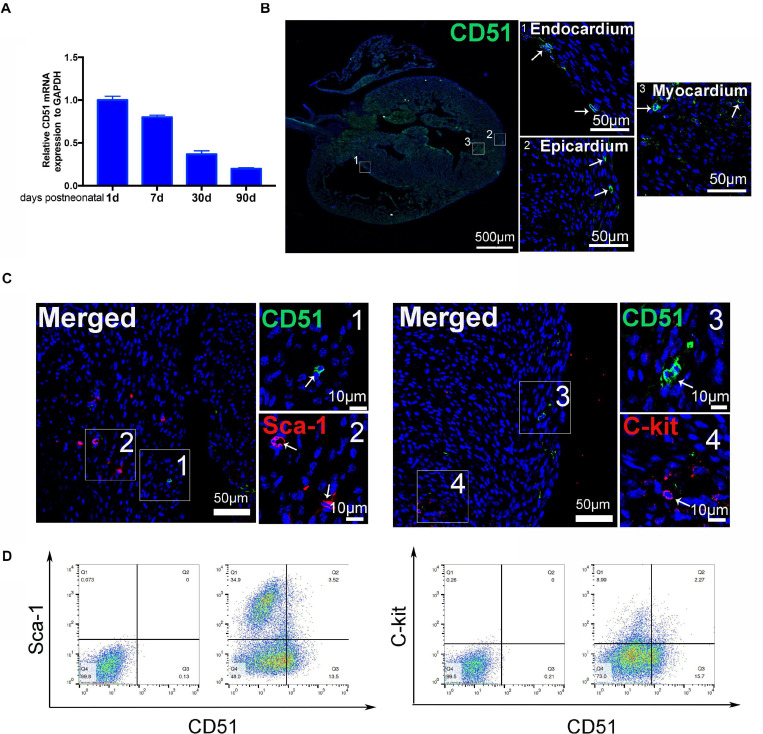
Characteristics of cardiac CD51 in mice. **(A)** mRNA expression of cardiac CD51 in mice of different ages was assessed by quantitative PCR, CD51 expression was decreasing with age, and data were normalized to GAPDH; values were presented as the mean ± SEM, *n* = 3. **(B**,**C)** Hearts from 7-day-old mice were stained with CD51 (green), Sca-1 (red), and C-kit (red); CD51-positive cells were located in all layers of the heart wall, including the endocardium, epicardium, and myocardium; few CD51-positive cells were co-localized with Sca-1- or C-kit-positive cells, nuclei were counterstained with DAPI (blue), and scale bars were marked in the figure. **(D)** Flow cytometry assessment of cardiac CD51-positive subpopulation with Sca-1- or C-kit-positive subpopulation in 7-day-old mice, and only a few cells simultaneous expressed two proteins.

We systematically evaluated the gene expression of CD51 in hearts during postnatal development. The data indicated that mRNA levels of cardiac CD51 were high in 1- and 7-day-old mice and intensely declined in 30- and 90-day-old mice (Figure 1A). Based on cardiac size, we selected 7-day-old neonatal mice to verify the location of cardiac CD51 by immunofluorescence stain. Our results show that CD51-positive cells were located in all layers of the heart wall, including the endocardium, epicardium, and myocardium (Figure 1B). Additionally, we performed immunofluorescence stain to investigate whether CD51^+^ cells express other interstitial cell fate markers. Notably, CD5 was not co-localized with Sca-1 or C-kit (Figure 1C). Moreover, data of flow cytometry proved that only a small part of Sca-1-positive cells could be stained by CD51 and also that a few C-kit-positive cells overlapped with CD51-positive cells (Figure 1D). Together, CD51-positive cells existed throughout the entire cardiac wall, decreased with age, and were distinct from the known Sca-1- or C-kit-positive cells.

### Comparison of Cardiac CD51^*pos*^ Cells and CD51^*neg*^ Cells From 7-Day-Old Mice

**FIGURE 2:**
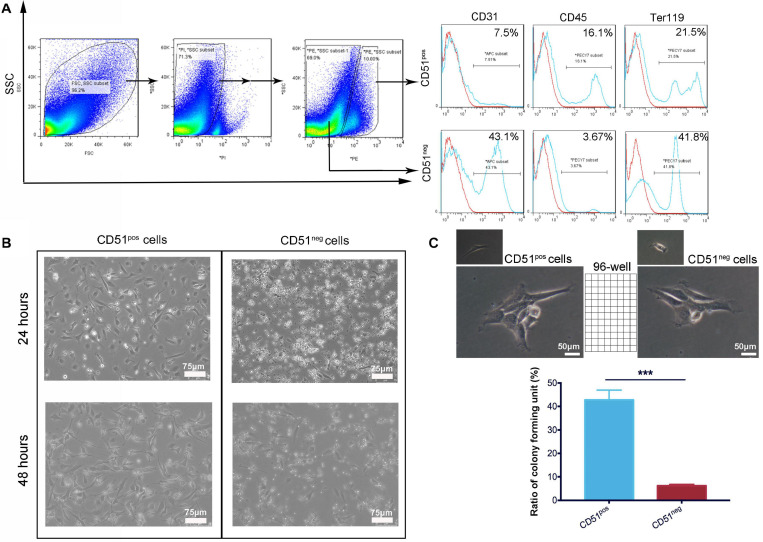
Comparison of cardiac CD51^*pos*^ cells and CD51^*neg*^ cells from 7-day-old mice. **(A)** Cardiac CD51^*pos*^ cells and CD51^*neg*^ cells were isolated by flow cytometry, and expression of CD31, CD45, and Ter119 in the CD51^*pos*^ and CD51^*neg*^ subpopulations was also analyzed; propidium iodide (PI) staining was used to discriminate dead cells. **(B)** Images of the cultured CD51^*pos*^ cells and CD51^*neg*^ cells, freshly sorted cells were cultured for 24 or 48 h in the medium. **(C)** Clone formation experiment: freshly sorted single cells were seeded in untreated 96-well plates and cultured for 7 days (one cell per well); representative images of replicated CD51^*pos*^ cells and CD51^*neg*^ cells were exhibited; structures with three or more cells in each well were considered to have clone-formation potential, and the positive ratio was calculated as follows: (number of colony-like structures/96 inoculated) × 100%; values were presented as the mean ± SEM, *n* = 3, ***P < 0.001. Scale bars were marked in the figure.

Considering that cardiac CD51 expression remained high in 7-day-old mice, we further characterized CD51^*pos*^ cells and CD51^*neg*^ cells in the heart tissues of 7-day-old mice *in vitro.* CD51 flow cytometry antibodies were used to classify the two types of cells, while antibodies against CD45, Ter119, and CD31 were used to analyze the overlapping subpopulations in hematopoietic, erythroid, and endothelial cell lineages, respectively. As shown in Figure 2A, the positive percentages of Ter119 and CD31 in CD51^*pos*^ cells (41.8 and 43.1%, respectively) were less than those in CD51^*neg*^ cells (21.5 and 7.5%, respectively), whereas expression of CD45 was higher in CD51^*pos*^ cells than in CD51^*neg*^ cells (16.1% vs. 3.67%). Then, we isolated the two types of cells using FACS and cultured them *in vitro* (Figure 2B). Almost all CD51^*pos*^ cells survived and adhered to the plate within 24 h, and they exhibited spindle morphology when cultured for 48 h. However, most of the CD51^*neg*^ cells died within 24 h, and the small part of surviving cells were also spindle-shaped. In addition, a single, freshly isolated CD51^*pos*^ cell was placed in each well of a 96-well plate with culture medium, and these colonies were observed after 7 days of culture. Notably, the ratio of colony-like structure in CD51^*pos*^ cell groups (42.77 ± 4.16%) was higher than that in CD51^*neg*^ cells (6.23 ± 0.59%) (Figure 2C).

### Isolation and Characterization of Cardiac CD51^+^cMSCs From 7-Day-Old Mice

**FIGURE 3:**
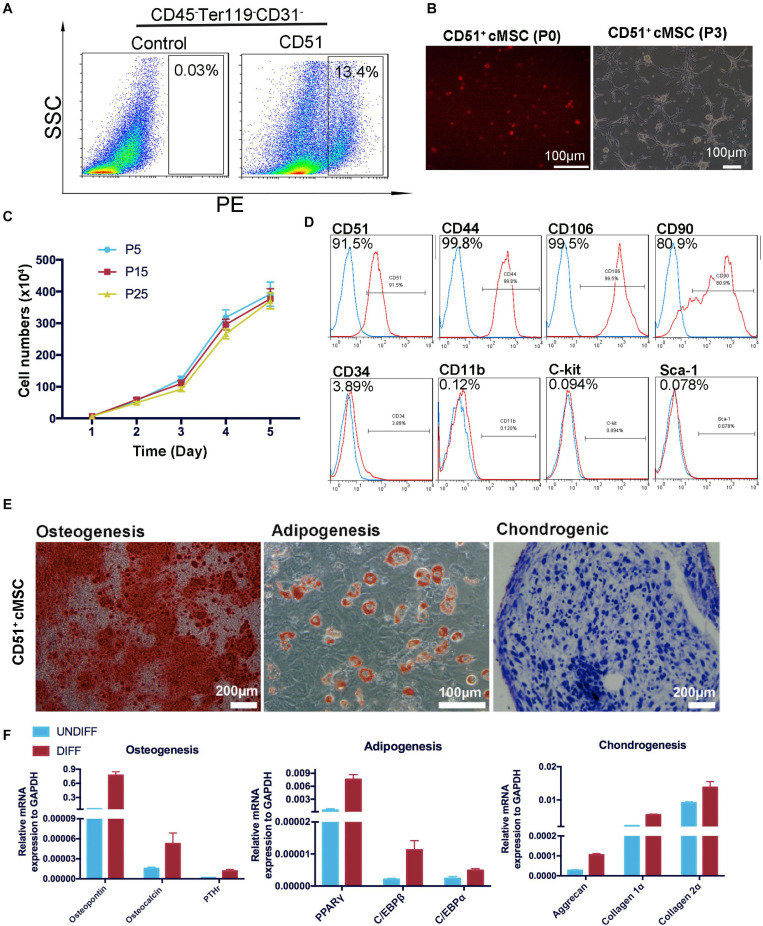
Isolation and identification of CD51^+^cMSCs. **(A)** Cardiac CD51^+^CD45^–^Terg119^–^CD31^–^ cells were isolated by flow cytometry, 7-day-old neonatal mice (30 mice per time) were used, and the positive rate of CD51-positive cells in CD45^–^Ter119^–^CD31^–^ populations was about 13.4%. **(B)** Images of the freshly isolated or subcultured CD51^+^cMSCs in plates: P0, passage 0; P3, passage 3. **(C)** Growth curves of CD51^+^cMSCs in different passages (P5, P15, and P25), cell numbers were calculated daily for 5 days, and data were presented as mean ± SEM, *n* = 3. **(D)** Detection of MSC-related marker expression in the cultured CD51^+^cMSCs (passage 5) using flow cytometry, the blue histogram was the negative control, and the right side of the red histogram indicated positive expression of the corresponding antibodies. **(E)** Multipotent differentiation of CD51^+^cMSCs (passage 5), osteogenesis (Alizarin Red), adipogenesis (Oil Red O), and chondrogenesis (Alcian blue), and scale bars have been marked in the figure. **(F)** Differentiated CD51^+^cMSCs were examined by quantitative PCR array to measure the specific genes of differentiated osteoblasts (osteopontin, osteocalcin, and PTHr), adipocytes (PPARγ, C/EBPβ, and C/EBPα), and chondrocytes (aggrecan, collagen 1α, and collagen 2α). Expression levels of each gene were compared to undifferentiated CD51^+^cMSCs, data were normalized to GAPDH, and values were shown as the mean ± SEM, *n* = 3.

Considering the advantages of CD51^*pos*^ cells, we sorted the CD51^+^CD45^–^Ter119^–^CD31^–^ subpopulation and identified its MSC-specific characterization *in vitro*. As shown in Figure 3A, the CD51^+^cMSCs accounted for approximately 13.4% of the total CD45^–^Ter119^–^CD31^–^ cells. Here, hematopoietic, erythroid, and endothelial cell lineages were excluded by being incubated with antibodies of CD45, Ter119, and CD31. The freshly isolated CD51^+^cMSCs were small and round after being suspended in the medium; they were adherent to the plates, exhibited a spindle-shaped morphology, and proliferated to form colony-like structures when subcultured to passage 3 (Figure 3B). The proliferative capacity of CD51^+^cMSCs at passages 5, 15, and 25 was stable when subcultured *in vitro* (Figure 3C). Further, we performed FACS to analyze the surface markers of CD51^+^cMSCs (passage 5). The subcultured CD51^+^cMSCs kept expressing CD51 markers (92 ± 2%). CD51^+^cMSCs positively expressed common MSC-specific surface markers, including CD44 (98 ± 1%), CD106 (98 ± 1%) and CD90 (83 ± 2%), but generally lowly expressed hematopoietic-related markers such as CD34 (3.6 ± 0.3%), CD11b (0.11 ± 0.03%), C-kit (0.09 ± 0.009%), and Sca-1 (0.067 ± 0.015%) (Figure 3D). To determine the plasticity of CD51^+^cMSCs, we cultured cells in the tri-lineage (osteocytes, adipocytes, and chondrocytes) differentiation mediums for 2–3 weeks. As shown in Figures 3E,F, CD51^+^cMSCs exhibited potential multipotent differentiation. They showed osteogenic, adipogenic, and chondrogenic differentiation properties when stained by Alizarin Red, Oil Red O, and Alcian blue, respectively. Related gene expression was observed by qPCR after induction in the specific culture medium. When compared to the undifferentiated cells, the specific genes were also significantly upregulated in the differentiated CD51^+^cMSCs, including osteogenic genes (osteopontin, osteocalcin, and PTHr), adipogenic genes (PPARγ, C/EBPβ, and C/EBPα) and chondrogenic genes (aggrecan, collagen 1α, and collagen 2α). Taken together, the cardiac CD51^+^cMSCs complied with MSC-specific criterion when cultured *in vitro*.

### CD51^+^cMSC Therapy Enhanced Cardiac Repair in Mice Post AMI

**FIGURE 4:**
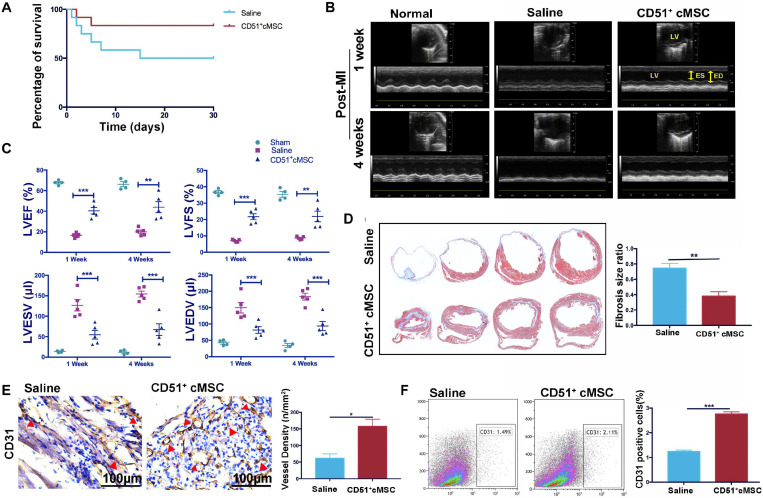
CD51^+^cMSC therapy enhanced cardiac repair in mice post AMI. **(A)** Analysis of the Kaplan–Meier survival curve using the log-rank test, χ^2^ = 2.762, *P* = 0.0965, *n* = 12 mice per group. **(B)** Representative M-mode echocardiography images of normal, saline-treated, and CD51^+^MSC-treated mice at 1 and 4 weeks post AMI. LV, left ventricle; ES, end systolic; ED, end diastolic. **(C)** Comparison of echocardiography parameters. LVEF, left ventricular ejection fraction; LVFS, left ventricular fractional shortening; LVESV, left ventricular end systolic volume; LVEDV, left ventricular end diastolic volume; data are presented as mean ± SEM, *n* = 4. **(D)** Assessment of myocardial fibrosis using Masson’s trichrome staining at 4 weeks post AMI; fibrosis size ratio was presented as a percentage of fibrosis length of the LV, *n* = 3. **(E)** Representative images of immunohistochemical staining with CD31 antibody in the infarcted myocardium at 2 weeks post AMI, the cone indicated the vessels, and values were presented as number of vessels per square meter, *n* = 3. **(F)** Analysis of cardiac CD31-positive cells in the infarcted area 2 weeks post AMI by flow cytometry, *n* = 3. Data in all panels are presented as mean ± SEM, *P < 0.05, **P < 0.01, and ***P < 0.001.

Endogenous stem cells were able to maintain tissue homeostasis and restore function after injury. To verify the repair function of CD51^+^cMSCs after AMI, CD51^+^cMSCs (1 × 10^5^) or saline were immediately transplanted into the infarct area of hearts. The number of surviving mice in the CD51^+^cMSC treatment group was high than that in the saline treatment group (Figure 4A). Furthermore, the function of LV was analyzed using M-mode echocardiography. The images show the short-axis view of LV, and parameters were recorded during the end systolic (ES) and end diastole (ED) phases (Figure 4B). Our results illustrated that the LVEF and LVFS in the saline groups (1 week: LVEF 16.61 ± 1.01% and LVFS 6.99 ± 0.36%; 4 weeks: LVEF 20.05 ± 1.62% and LVFS 8.70 ± 0.41%) were significantly deteriorated compared with that in CD51^+^MSC-treated mice (1 week: LVEF 40.45 ± 3.15% and LVFS 21.71 ± 1.83%; 4 weeks: LVEF 43.96 ± 5.39% and LVFS 21.84 ± 3.23%) at 1 and 4 weeks post AMI. Meanwhile, the values for LVESV (μl) and LVEDV (μl) were obviously reduced in CD51^+^MSC-treated mice (1 week: LVESV 54.81 ± 10.555 and LVEDV 81.66 ± 10.22; 4 weeks: LVESV 67.22 ± 14.16 and LVEDV 93.89 ± 13.74) compared with those in saline-treated mice (1 week: LVESV 126.7 ± 13.95 and LVEDV 149.3 ± 16.05; 4 weeks: LVESV 154.3 ± 7.1 and LVEDV 183.6 ± 10.30) (Figure 4C). At 4 weeks after AMI, the infarcted area of the LV became very thin and was totally replaced by fully differentiated scar tissues. Therefore, mice were sacrificed at 4 weeks after AMI, and hearts were collected for Masson’s trichrome stain to analyze the fibrosis size. To clearly reflect the degree of ventricular remodeling among groups, we used length parameter as recommended by Liaoyan (Liao et al., 2020) which was calculated by the ratio of the length of the infarct band to the total length of the LV. Notably, the ratio of the infarcted area was markedly reduced in the CD51^+^MSC treatment group (35.55 ± 3.63%) compared with that in the saline-treated control mice (73.58 ± 6.34%) (Figure 4D).

### CD51^+^cMSC-Based Therapy Enhanced Angiogenesis After AMI

Angiogenesis plays an important role in the remodeling process, which provides oxygen and nutrients to support tissue growth and healing after ischemic heart disease (Badimon and Borrell, 2018). We measured the number of capillaries in the infarct zone by staining CD31 at 2 weeks post AMI. In the CD51^+^MSC group, the capillary density was significantly increased compared with that in the saline group (Figure 4E). Additionally, the proportion of cardiac CD31^+^ cells in the infarcted area significantly increased in the CD51^+^MSC transplantation groups when compared with the saline-treated group (Figure 4F). Taken together, CD51^+^cMSC transplantation effectively enhanced angiogenesis in the infarcted myocardium.

### SCF Secreted by CD51^+^cMSCs Functioned as a Paracrine Protein

**FIGURE 5:**
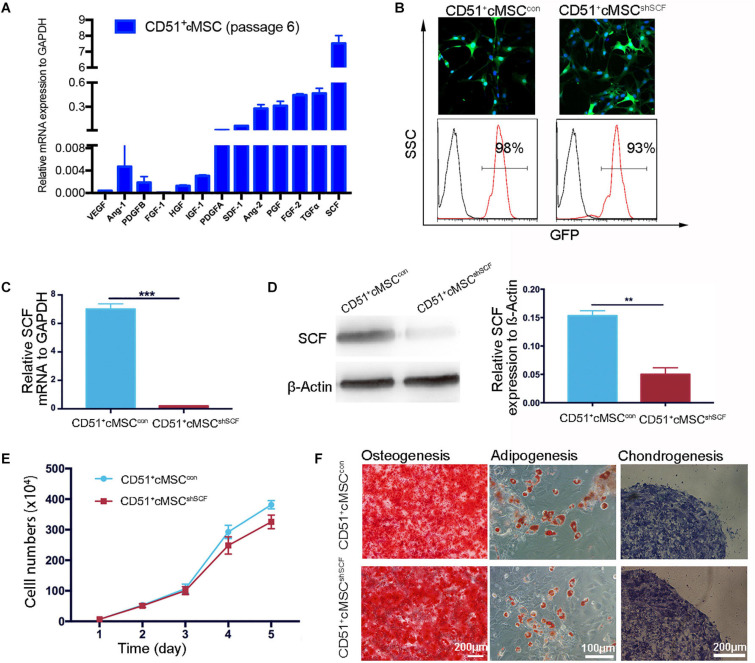
Downregulation of functional SCF in CD51^+^cMSCs. **(A)** Assessment of the angiogenesis-related mRNA expression in CD51^+^cMSCs (passage 6) by qPCR; SCF showed the highest expression among the tested genes, *n* = 3. **(B)** Lentivirus infection efficiency was measured via GFP expression using a microscope and flow cytometry; CD51^+^cMSC^*shSCF*^: the lentiviral vector carrying the shRNA; CD51^+^cMSC^*con*^: the lentiviral carrying the mock vector. **(C)** Detection of SCF mRNA expression in lentiviral-infected cells using qPCR 72 h post infection, *n* = 3. **(D)** Estimation of SCF protein in lentiviral-infected cells using western blotting 4 days after infection, *n* = 3. **(E)** The proliferative curve of the cultured CD51^+^cMSC^*shSCF*^ and CD51^+^cMSC^*con*^; cells were in passage 8, *n* = 5. **(F)** Multipotent differentiation of CD51^+^cMSC^*con*^ and CD51^+^cMSC^*shSCF*^, osteogenesis (Alizarin Red), adipogenesis (Oil Red O), and chondrogenesis (Alcian blue); scale bars have been marked in the figure. Data in all panels are presented as means ± SEM, **P < 0.01 and ***P < 0.001.

Based on accumulating evidence, stem cells enhance endogenous repair and angiogenesis processes by releasing paracrine factors. As angiogenesis effects were observed in our study, we further examined the expression of previously reported paracrine factors in CD51^+^cMSCs: VEGF, Ang-1, PDGFB, FGF-1, HGF, IGF-1, PDGFA, SDF-1, Ang-2, PGF, TGFα, and SCF. Remarkably, SCF was expressed at the highest levels in the CD51^+^cMSCs (Figure 5A). To further investigate the role of SCF, lentiviral plasmid DNA containing SCF-shRNA with a GFP expression cassette was transfected into CD51^+^cMSCs to silence SCF expression. Data of FACS indicated that the percentages of GFP-positive cells in the SCF interference group (CD51^+^cMSC^*shSCF*^) and mock vector control group (CD51^+^cMSC^*con*^) were 93 ± 2.5% and 98 ± 0.9%, respectively (Figure 5B). The mRNA level of SCF was downregulated by ∼90% in the CD51^+^cMSC^*shSCF*^ group when compared to the CD51^+^cMSC^*con*^ group (Figure 5C). Consistently, the protein level of SCF notably decreased by ∼70% in the CD51^+^cMSC^*shSCF*^ group (Figure 5D). The proliferative capacities of CD51^+^cMSC^*shSCF*^ and CD51^+^cMSC^*con*^ were similar (Figure 5E). Also, the potential to differentiate into osteogenic, adipogenic, and chondrogenic lineage phenotypes was preserved after SCF was silenced (Figure 5F). Thus, CD51^+^cMSCs expressed high levels of SCF, and SCF silence did not alter the proliferation and differentiation capacity in CD51^+^cMSCs.

### CD51^+^cMSC Therapy Improved Heart Function Through SCF-Mediated Angiogenesis *in vivo*

**FIGURE 6:**
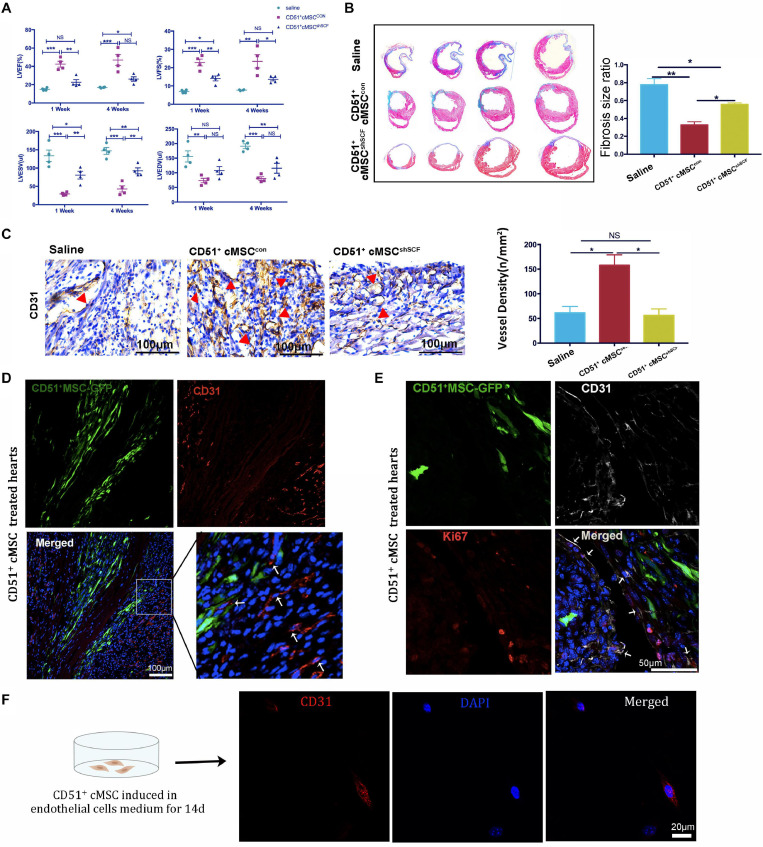
CD51^+^cMSC therapy improved heart function through SCF-mediated angiogenesis *in vivo*, *n* = 12 mice per group. **(A)** Analysis of echocardiography parameters at 1 and 4 weeks after treatment; LVEF, left ventricular ejection fraction; LVFS, left ventricular fractional shortening; LVESV, left ventricular end systolic volume; LVEDV, left ventricular end diastolic volume, *n* = 4. **(B)** Assessment of myocardial fibrosis using Masson’s trichrome staining 4 weeks after treatment; the fibrosis size ratio was presented as percentage of fibrosis length of the LV, *n* = 3. **(C)** Images of immunohistochemical staining with CD31 antibody in the infarcted myocardium 2 weeks after injury, the cone indicated the vessels, and values were presented as numbers of vessels per square meter, *n* = 3. **(D)** Representative images of immunofluorescence staining with CD31 antibody in the infarcted myocardium 7 days after treatment: GFP-positive CD51^+^cMSCs (green); CD31 (red); DAPI (blue). **(E)** Immunofluorescence staining of the heart with AMI 1 week after CD51^+^cMSC treatment: CD51^+^cMSCs (green), CD31 (white), Ki67 (red), DAPI (blue); arrows indicated co-localization of CD31 and Ki67, scale bars 50 μm. Data in all panels are presented as means ± SEM, NS: *P* > 0.05; *P < 0.05, **P < 0.01, and ***P < 0.001. **(F)** CD51^+^MSCs were cultured in the endothelial cell medium for 14 days and stained with CD31.

To investigate whether CD51^+^cMSC-secreted SCF contributes to the cardioprotective effect by angiogenesis, CD51^+^cMSCs^*shSCF*^ (1 × 10^5^ cells per mouse), CD51^+^cMSCs^*con*^ (1 × 10^5^ cells per mouse), or saline was immediately transplanted into the infarcted area after AMI. The heart function was assessed at 1 and 4 weeks after treatment (Figure 6A). The LVEF did not significantly restore at 1 week after the CD51^+^cMSC^*shSCF*^ treatment compared with the saline treatment. LVFS had no notable difference between CD51^+^cMSC^*shSCF*^ treatment and saline groups at 4 weeks after treatment. Moreover, the parameters of LVEF, LVFS, and LVESV were deteriorated in the CD51^+^cMSC^*shSCF*^ treatment groups when compared with CD51^+^cMSCs^*con*^ treatment groups. Data of Masson’s trichrome stain showed that the cardiac fibrosis ratio significantly increased in the CD51^+^cMSC^*shSCF*^-treated mice (53.48 ± 2.57%) and saline-treated control mice (73.58 ± 6.34%) when compared with CD51^+^cMSC^*con*^-treated mice (35.55 ± 3.64%) (Figure 6B). Meanwhile, we examined the potential of angiogenesis *in vivo* after AMI. Two weeks after therapy, the number of the blood vessels in the infarcted regions significantly decreased in CD51^+^cMSC^*shSCF*^-treated mice (56 ± 13), when compared with CD51^+^cMSCs^*con*^-treated mice (157 ± 21) (Figure 6C). Furthermore, we transplanted GFP-labeled CD51^+^cMSCs into the infarcted hearts and found that the CD31^+^ endothelial cells abundantly surrounded the transplanted CD51^+^cMSCs at 7 days after therapy (Figure 6D). Interestingly, a moderate number of endothelial cells were in proliferative fate around CD51^+^cMSCs (Figure 6E). Also, CD51^+^cMSCs rarely expressed CD31 after being induced in the endothelial medium for 14 days (Figure 6F). Thus, CD51^+^cMSC-secreted SCF played a vital role in angiogenesis of the injured heart, which induced cardiac repair in post-AMI mice.

### SCF Downregulation in CD51^+^cMSCs Decreased Function of Endothelial Cells *in vitro*

**FIGURE 7:**
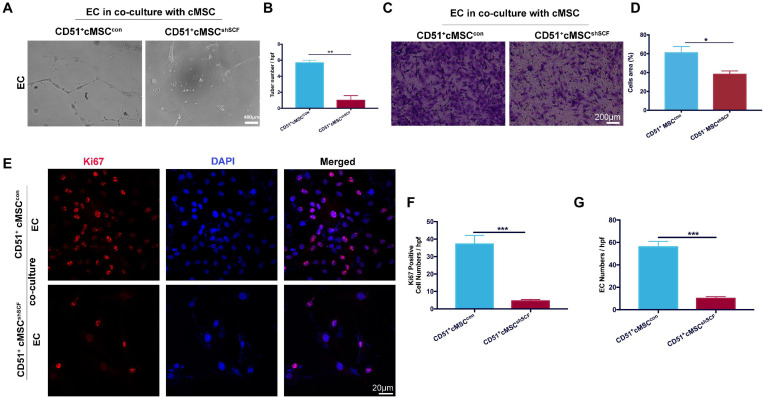
SCF downregulation in CD51^+^cMSCs decreased function of endothelial cells *in vitro*. **(A**,**B)** Tube formation of endothelial cells on Matrigel-coated dishes when co-cultured with CD51^+^cMSC^*shSCF*^, or CD51^+^cMSC^*con*^ under hypoxia conditions; number of tubes per high-power field (hpf) was analyzed, *n* = 3. **(C**,**D)** Transwell array: migration of endothelial cells when cocultured with CD51^+^cMSC^*shSCF*^, or CD51^+^cMSC^*con*^ under hypoxia conditions, endothelial cells were stained by crystal violet, and area percentage of cells in each hpf area was analyzed, *n* = 5. **(E**–**G)** Representative images of immunofluorescence staining with Ki67 antibody in endothelial cells after being cocultured with CD51^+^cMSC^*shSCF*^, or CD51^+^cMSC^*con*^ under hypoxia conditions: Ki67 (red), DAPI (blue); numbers of Ki67-positive cells and total endothelial cells per field were analyzed, *n* = 5. EC, endothelial cells. Data in all panels were presented as means ± SEM, *P < 0.05, **P < 0.01, and ***P < 0.001.

To confirm the role of SCF from CD51^+^cMSCs in the angiogenesis potential of endothelial cells, we performed coculture experiments under hypoxia conditions *in vitro*: endothelial cells cocultured with CD51^+^cMSC^*shSCF*^ and CD51^+^cMSC^*con*^. The number of endothelial tubular structures in the CD51^+^cMSC^*shSCF*^ group (1.0 ± 0.57) was lower than that of the CD51^+^cMSC^*con*^ group (5.6 ± 0.33) (Figures 7A,B). In addition, more endothelial cells migrated from the upper membrane to the lower surface of the filter in the CD51^+^cMSC^*con*^ coculture system than that in the CD51^+^cMSC^*shSCF*^ system (Figures 7C,D). Finally, we observed the proliferative potential of endothelial cells by stain Ki67 after coculture for 48 h (Figure 7E). The number of Ki67-positive cells (counted per field) was 37.2 ± 4.95 in the CD51^+^cMSC^*con*^ group, which was noticeably elevated when compared to the CD51^+^cMSC^*shSCF*^ group (4.6 ± 0.81) (Figure 7F). The total number of endothelial cells was 56 ± 5.02 per field in the CD51^+^cMSC^*con*^ group, which was significantly increased when compared to the CD51^+^cMSC^*shSCF*^ group (10 ± 1.581 per field) (Figure 7G). In summary, SCF secreted by CD51^+^cMSCs enhanced the function and proliferative capacity of endothelial cells.

## Discussion

Considering resident MSCs are promising stem cell sources for the treatment of tissue-specific diseases, optimal markers of cardiac resident MSCs must be well identified. In this study, CD51 was served as a novel marker to identify and isolate cardiac MSCs. We confirmed the MSC-specific characteristics of CD51^+^ cMSCs *in vitro*: proliferative capacity, multi-differentiation potential, and expression of MSCs-related markers. CD51^+^cMSC transplantation moderately improved heart function and attenuated fibrosis in post-AMI mice through SCF-mediated angiogenesis. Upon silencing the SCF gene, the pro-angiogenesis effect decreased both *in vivo* and *in vitro*. In summary, our findings provided new insights into the identification and application of cardiac resident MSCs.

MSCs *in situ* participated in regulating their specific environmental niche by maintaining its stability and adjusting gene expression at different levels (Tsai et al., 2007). We postulated that cardiac resident MSCs have distinct advantages to treat AMI without significant heterogeneity. Recently, W8B2-marked cells, derived from adult human atrial appendages, possessed MSC properties and secreted a variety of cytokines which were associated with angiogenesis, but their proangiogenic effects disappeared when transplanted as a single-cell suspension (Zhang et al., 2015). To date, cardiac MSCs have not been clearly identified due to lack of identification markers. Importantly, we observed CD51 as a promising marker of resident MSCs in hearts, and its expression was moderate in neonatal mice but decreased in adult mice. We also confirmed by flow cytometry analysis that the CD51 expression decreased with age in mice (data not shown). Consistently, Sandra Pinho has reported that the positive percentage of CD51^+^ stromal cells was ∼6% in the fetal human bone marrow, which was higher than that in adult bone marrow (a lower frequency <1%) (Pinho et al., 2013). Also, CD51 was essential in controlling the septation remodeling of the heart and outflow tract during early embryonic development (at ∼E9.5 in mice) (Turner et al., 2015). Jun Tang et al. have reported that cardiac Sca-1-positive cells contributed to cardiac endothelial cells and fibroblasts (Tang et al., 2018). He Lingjuan et al. demonstrated that the non-cardiomyocytes of C-kit-positive cells matched all hematopoietic cells and coronary endothelial cells in hearts of mice (He et al., 2019). Here, the CD51^+^ cells were a different subpopulation from the Sca-1-positive cells or C-kit cells. Unlike the heterogeneous CD51^*neg*^ cells, CD51^*pos*^ cells were also distinct from endothelial cells and erythrocytes. In addition, the CD51^*pos*^ cells in neonatal hearts have no specific positional relationship with endothelial cells when assessed by immunofluorescence histology (data not shown). Therefore, CD51 was employed as a marker to isolate cardiac MSCs.

We showed that the positive percentage of CD51^+^cMSCs from neonatal mice was moderate, while Milena B. Furtado reported that CD51 stained >95% of adult primary cardiac fibroblasts cultured for 5–7 days in the DMEM high-glucose medium supplemented with 10% FBS (Furtado et al., 2014). The CD51 ratio in our study was tested from tissue samples, but Milena B. Furtado evaluated it from cultured fibroblast samples; the different ratio is not contradictory: (1) The cardiac CD51-positive cells were partially overlapped with hematopoietic, erythroid, and endothelial cell lineages, and the number of CD51^+^cMSCs further decreased after these cell lineages were excluded; (2) CD51 is an integrin, which promotes adhesion and signal transduction capabilities. Adherent growth cells are prone to express CD51 when cultured in medium supplemented with 10% FBS; CD51 expression of cardiac fibroblasts could be induced under this culture condition. Importantly, CD51^+^cMSCs were able to propagate *in vitro* for months and maintained vigorous long-term proliferative capacity for at least 35 passages. Except for their multi-differential potential, CD51^+^cMSCs also expressed CD106, CD44, and Sca-1, but barely CD45, CD34, and CD11b-1. These properties were consistent with the criteria for defining minimal MSC from the International Society for Cellular. All cells were adherent and spindle-shaped when cultured *in vitro*, CD51^+^cMSCs were prone to form colony-like structures, and fibroblasts were scattered. Notably, the quantitative RT-PCR analysis indicated that typical fibroblast-related gene expression (DDR2: discoidin domain receptor 2, S100A4: S100 calcium-binding protein, and CD140a) in CD51^+^cMSCs was obviously lower than that in fibroblasts. Several studies reported the similarity and differences observed between MSCs and fibroblasts in several gene expressions (Covas et al., 2008). Importantly, increased evidence indicated that fibroblasts are in fact aged MSCs and that the two cells are the same, but this still requires further research (Soundararajan and Kannan, 2018). As previously reported, the *bona fide* MSCs marked by CD51 and PDGFRα were capable of differentiating into multiple lineages (Pinho et al., 2013). James J.H. Chong has clarified that PDGFRα defined all cardiac-resident colony-forming units—fibroblast originating from the proepicardium (in a perivascular position) in adult hearts (Chong et al., 2011). In our study, CD51^+^ and PDGFRα^+^ cells partially overlapped, so we further classified CD45^–^CD31^–^Ter119^–+^cells, and they were separated into three distinct fractions: CD51^+^PDGFRα^–^MSCs, CD51^+^PDGFRα^+^MSCs, and CD51^–^PDGFRα^+^MSCs. When cells were cultured in the indicated medium, CD51^+^PDGFRα^–^ MSCs and CD51^+^PDGFRα^+^ MSCs kept good growth vitality even when propagated to > 35 passages, but CD51^–^PDGFRα^+^ MSC had slow growth after being propagated to passage 5; the osteogenesis and adipogenesis capacities of PDGFRα^+^CD51^–^ cells were also decreased. Additionally, ∼40% freshly sorted CD51^+^cMSCs expressed PDGFRα (Supplementary Figure 1A), and the ratio of PDGFRα was similar in the subcultured CD51^+^cMSCs (data not shown). Collectively, these results suggest that CD51 might be an earlier marker for cardiac MSCs compared with PDGFRα.

MSCs serve as a potential strategy to repair damaged cardiac tissues and restore heart function after AMI (Kim et al., 2015). However, the donor bone marrow-derived MSCs were rapidly lost in the recipients, which potentially limited their therapeutic efficacy (Imanishi et al., 2008). According to Shimomura et al. (2010) allogeneic synovial MSCs could specifically target cartilage and repair tissue injury (Shimomura et al., 2010). We delivered CD51^+^cMSCs to hearts of post-AMI mice via myocardial injection and notably confirmed their therapeutic efficiency. The main pathophysiology of AMI is cardiomyocyte death and microvascular dysfunction (Heusch and Gersh, 2017). Corresponding to the pathophysiological process, the therapeutic actions of MSCs were mainly mediated by two mechanisms: anti-inflammatory and pro-angiogenesis (Teng et al., 2015; Tao et al., 2016; Eirin et al., 2017). MSCs facilitated heart contractility and myocardial blood flow by reducing fibrosis, promoting angiogenesis, and decreasing cell death after heart injury (Quevedo et al., 2009; Golpanian et al., 2016). Angiogenesis around transplanted CD51^+^cMSCs in the infarcted area was obviously increased after treatment. In addition, we confirmed the pro-angiogenic function of cardiac CD51^+^ cMSCs *in vitro* in homologous cardiac endothelial cells and also in human umbilical vein endothelial cells. Therefore, CD51^+^cMSC therapy enhanced the microvascular function by modulating angiogenesis.

MSCs contributed to angiogenesis through paracrine signaling (Silva et al., 2005; Gnecchi et al., 2006). Also, Ranganath et al. (2012) emphasized that the therapeutic potential of MSCs was mediated by a large amount of secreted factors (Ranganath et al., 2012). We analyzed the gene expression of these pro-angiogenesis factors and found that SCF was highly expressed in CD51^+^cMSCs. Indeed, the pro-angiogenesis effect of SCF was also emphasized by D Blaise and Lixin Sun on patients (Blaise et al., 2000; Sun et al., 2006). Consistently, we confirmed that CD51^+^cMSCs enhanced tubulogenesis, migration, and proliferation of endothelial cells *in vitro* via SCF. Shafie Fazel illustrated that implantation of bone marrow MSCs increased SCF levels in the hearts and promoted myocardial angiogenesis, and this angiogenesis benefit was enhanced by SCF overproduction (Fazel et al., 2005). Similarly, we observed that CD51^+^cMSCs contributed to angiogenesis in the injured area of hearts mainly through SCF paracrine. When SCF was knocked down, the angiogenesis effect of CD51^+^cMSCs intensely declined, and the remaining therapeutic effects were likely provided by other secreted paracrine factors, such as TGFα, FGF-2, PGF, and Ang-2. Moreover, a clinical trial implemented by Wigren et al. (2016) suggested that the SCF decrease complied with the incidence of coronary events, and SCF was considered a therapeutic strategy to treat cardiovascular disease (Wigren et al., 2016). SCF downstream signaling pathways are very complex. Several studies reported that SCF controls physiological and pathological processes in tissues via the Wnt/β-catenin signaling pathway, Ras/Erk signal transduction pathway, PI3K signaling pathway, Src kinase signaling pathway, JAK/STAT signaling pathway, and PLC-γ pathway (Yaniz-Galende et al., 2012; Liang et al., 2013). We would perform in-depth studies to determine the exact signaling transduction pathway in the future. Also, limitations existed in this research: the source of cells in clinical applications is relatively limited, most of the cardiac tissues were obtained from the right atrium during surgery, and only allogeneic stem cells can be used for treatment. To solve this problem, we prepared to detect the functional regulatory factors in CD51^+^cMSCs and enhance this factor in bone marrow- or umbilical cord-derived stem cells by genetic engineering technologies.

## Conclusion

The cardiac-derived CD51^+^cMSCs represented a potential MSC subpopulation *in situ*, which is equipped with a self-renewal ability and multi-lineage differentiation potential and expressed typical MSC markers. CD51^+^cMSC therapy improved heart function and decreased fibrosis in post-AMI mice, and SCF-mediated angiogenesis participated in the repair process. CD51 is a novel marker for cardiac MSCs, which makes CD51^+^cMSCs a promising cell source to treat AMI.

## Data Availability Statement

The original contributions presented in the study are included in the article/Supplementary Material, further inquiries can be directed to the corresponding author/s.

## Ethics Statement

The animal study was reviewed and approved by Animal Ethical and Welfare Committee of Sun Yat-sen University.

## Author Contributions

MJ and CP: conception and design, data analysis and interpretation, manuscript writing, final approval of manuscript, and financial support. DX: cell and animal experiments, immunofluorescence stain, and manuscript writing. YC: data analysis. YL and DL: echocardiography. KY and ZC: flow cytometry. WL and SZ: assembly of data. GD and BW: myocardial infarction model establishment. All authors contributed to the article and approved the submitted version.

## Conflict of Interest

YL is now employed by company Shenzhen Beike Biotechnology Co., Ltd. The remaining authors declare that the research was conducted in the absence of any commercial or financial relationships that could be construed as a potential conflict of interest.

## Supplementary Material

Supplementary Figure 1 Isolation and characterization of the three subpopulations. **(A)** Isolation of CD51^+^PDGFRα^–^ MSCs, CD51^+^PDGFRα^+^ MSCs, and CD51^–^PDGFRα^+^ MSCs using FACS; the positive rates of CD51^+^PDGFRα^+^, CD51^+^PDGFRα^–^, and CD51^–^PDGFRα^+^ were about 2.09, 3.89, and 3.6%, respectively. Fifty neonatal C57BL/6 mice were used. **(B)** Morphology of the three subpopulations when cultured for 5 days *in vitro* after fresh isolation. **(C)** Osteogenic (Alizarin Red) and adipogenic (Oil Red O) differentiation of three subpopulations at passage 1.
