# Supplementary material for: Cardiac Derived CD51-Positive Mesenchymal Stem Cells Enhance the Cardiac Repair Through SCF-Mediated Angiogenesis in Mice With Myocardial Infarction
Source: Front Cell Dev Biol. 2021 Apr 21;9:642533. doi: 10.3389/fcell.2021.642533 (PMC8098770; doi:10.3389/fcell.2021.642533)
Supplement: Supplementary Figure 1 — Isolation and characterization of the three subpopulations. (A) Isolation of CD51+PDGFRα– MSCs, CD51+PDGFRα+ MSCs, and CD51–PDGFRα+ MSCs using FACS; the positive rates of CD51+PDGFRα+, CD51+PDGFRα–, and CD51–PDGFRα+ were about 2.09, 3.89, and 3.6%, respectively. Fifty neonatal C57BL/6 mice were used. (B) Morphology of the three subpopulations when cultured for 5 days in vitro after fresh isolation. (C) Osteogenic (Alizarin Red) and adipogenic (Oil Red O) differentiation of three subpopulations at passage 1. [file Data_Sheet_1.docx]

**Supplement**

**Supplement figures**


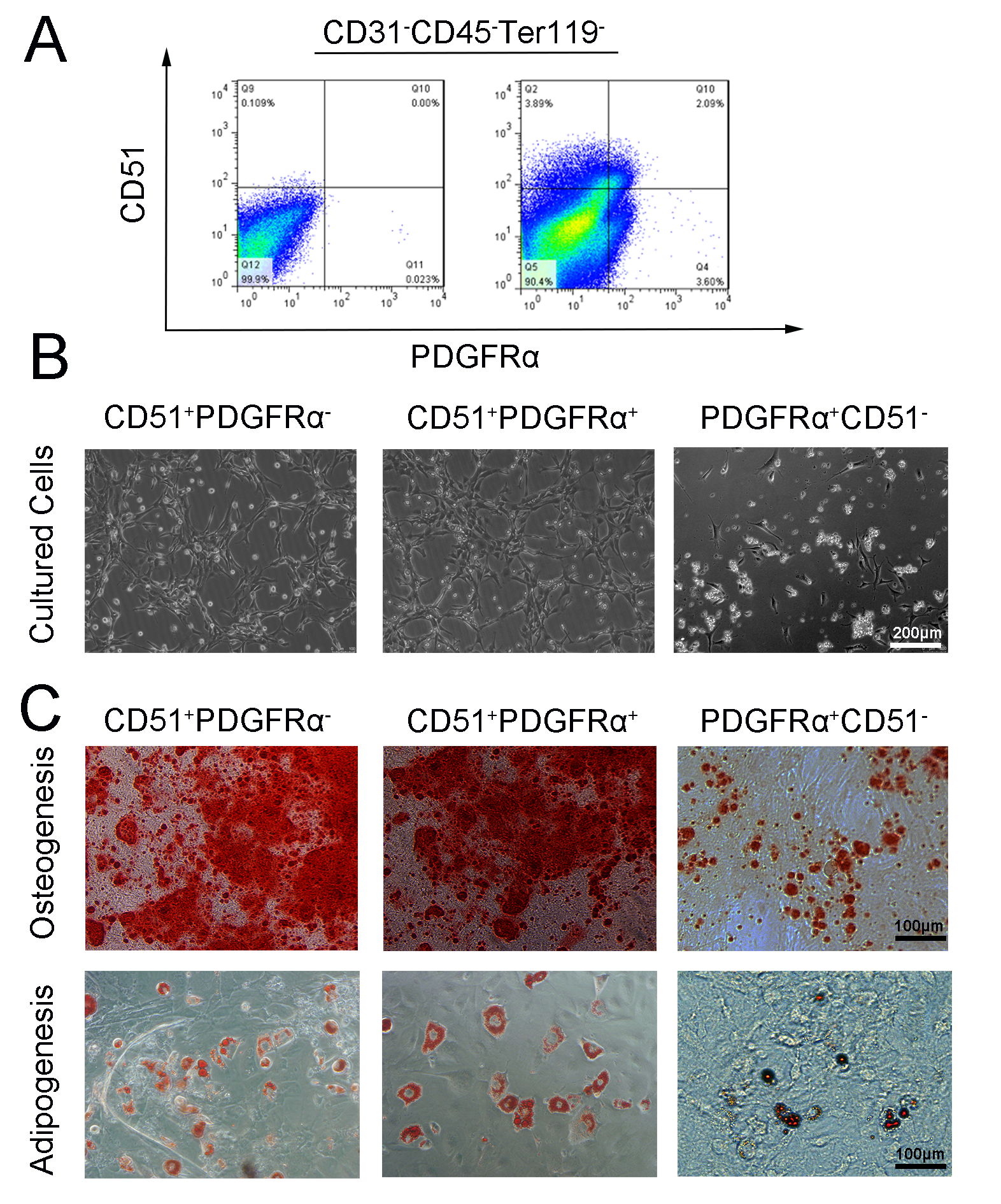


**Figure S1. Isolation and characterization of the three subpopulations.** **A**. Isolation of CD51^+^PDGFRα^-^MSCs, CD51^+^PDGFR­­α^+^MSCs, and CD51^-^PDGFR­­α^+^MSCs using FACS, the positive rate of CD51+ PDGFRα+; CD51+ PDGFRα-; CD51- PDGFRα+ were about 2.09%, 3.89%, 3.6% respectively. 50 neonatal C57BL/6 mice were used. **B**. Morphology of the three subpopulations when cultured 5d in vitro after freshly isolation. **C**. Osteogenic (alizarin red) and adipogenic (oil red O) differentiation of three subpopulations at passage 1.
